# Investigating the impact of peer supplemental instruction on underprepared and historically underserved students in introductory STEM courses

**DOI:** 10.1186/s40594-022-00372-w

**Published:** 2022-09-05

**Authors:** Chantelle Anfuso, Judy Awong-Taylor, Jamye Curry Savage, Cynthia Johnson, Tirza Leader, Katherine Pinzon, Benjamin Shepler, Cindy Achat-Mendes

**Affiliations:** Georgia Gwinnett College, Lawrenceville, GA 30043 United States

**Keywords:** Gateway courses, Supplemental instruction, Academic preparedness, Historically underserved, Equity, Diversity, Student success, STEM education

## Abstract

**Background:**

Supplemental instruction (SI) is a well-established mode of direct academic support, used in a wide variety of courses. Some reports have indicated that SI and similar peer-led academic support models particularly benefit students identifying with historically underserved racial/ethnic groups in STEM. However, these studies have not explicitly examined the role of prior academic experiences, an important consideration in college success. We report on the impact of a modified SI model, Peer Supplemental Instruction (PSI), on student success in introductory STEM courses at a diverse access institution. This study focuses on PSI’s impact on the academic performance of students identifying with historically underserved racial/ethnic groups, while also considering the effects of prior academic experiences.

**Results:**

Data were aggregated for nine courses over five semesters to produce a robust data set (*n* = 1789). PSI attendees were representative of the overall student population in terms of previous academic experiences/performance (as determined by high school GPA) and self-identified racial/ethnic demographics. Frequent PSI attendance was correlated with a significant increase in AB rates (average increase of 29.0 percentage points) and reduction in DFW rates (average decrease of 26.1 percentage points) when comparing students who attended 10 + vs. 1–2 PSI sessions. Overall, students identifying as Black/African American received the largest benefit from PSI. These students experienced a significant increase in their final course GPA when attending as few as 3–5 PSI sessions, and exhibited the largest increase in AB rates (from 28.7 to 60.5%) and decrease in DFW rates (from 47.1 to 14.8%) when comparing students who attended 10 + vs. 1–2 sessions. However, students with similar HS GPAs experienced similar benefits from PSI, regardless of self-identified race/ethnicity.

**Conclusions:**

The data presented here suggest that PSI particularly benefitted underprepared students in their introductory STEM courses. Since students identifying with historically underserved racial/ethnic groups have traditionally had inequitable K–12 educational experiences, they enter college less prepared on average, and thus particularly benefit from PSI. PSI, in conjunction with additional strategies, may be a useful tool to help rectify the results of systemic educational inequities for students identifying with historically underserved racial/ethnic groups.

## Introduction

It has been widely recognized in recent years that individuals identifying as Black/African American, Hispanic/Latino, and other persons of color are underrepresented in the STEM workforce and academia in the U.S. According to recent statistics, individuals identifying as Black/African American and Hispanic/Latino comprised 14.2% and 21.3% of the U.S. population aged 20–34, but were awarded only 8.7% and 16.3% of STEM bachelor’s degrees in 2019 and comprised only 5.1% and 7.6% of the STEM workforce, respectively (National Science Board, National Science Foundation, 2022). A major focus of addressing this discrepancy has been to increase STEM enrollment and retention for college students identifying with these historically underserved (HU) racial/ethnic groups. However, data show that even when students identifying with these groups choose STEM majors, they are more likely to struggle in STEM programs, change to non-STEM majors, and/or leave college (President’s Council of Advisors on Science & Technology, 2012). It is important to improve educational outcomes for students identifying with HU groups in STEM to achieve representative diversity in the nation's STEM workforce.

Efforts to improve educational outcomes for students identifying with HU groups often include the use of High Impact Practices (HIPs). These approaches include well-tested, evidence-based teaching practices that have been shown to be beneficial for all students, especially in STEM retention, progression, and graduation rates (Brownell & Swaner, 2010; Conefrey, 2018; Finley & McNair, 2013; Kuh, 2008; Kuh & O'Donnell, 2013; Peters et al., 2019; Thomas et al., 2018). Participation in HIPs, however, is inequitable; first generation students and students identifying as Black/African American or Hispanic/Latino are less likely to have access to HIPs throughout their educational careers (Conefrey, 2018; Kuh et al., 2017). Assessing which students have access to HIPs, in addition to the impact of these practices, is therefore of interest in designing approaches to improve educational outcomes for students identifying with HU groups.

One of the 11 HIPs originally established by Kuh (2008) is Collaborative Assignments and Projects, which can take the form of study groups or peer-led study sessions. One such model is supplemental instruction (SI), a well-established mode of peer-led direct academic support that is used internationally to promote student success in a wide variety of courses and disciplines. The School of Science and Technology at Georgia Gwinnett College (GGC) has developed and implemented a modified supplemental instruction (SI) program, termed Peer Supplemental Instruction (PSI), which we reported on previously (Achat-Mendes et al., 2020). Our initial study found that frequent PSI participation correlated with higher course grades and lower DFW rates. PSI appeared to be particularly beneficial for academically disadvantaged students, as determined by their incoming high school grade point average (HS GPA): students with lower HS GPAs experienced gains of approximately one letter grade with frequent PSI attendance, while students with high HS GPAs (> 3.5) saw no significant increase in their final course grades (Achat-Mendes et al., 2020). Here, we expand upon our previous work to offer new insights into how this program can benefit students identifying with HU populations in particular, and how the impact of PSI on these students can be affected by their prior academic experiences.

## Background

### Supplemental instruction (SI)

Supplemental instruction (SI) is based on the constructivist learning theory, which maintains that student learners must collaboratively and actively build their own knowledge base, rather than passively take in information from instructors (Zerger, 2008). In this academic support model, peer “SI Leaders” facilitate learning in sessions outside of regular class hours. Students enrolled in the course may attend for extra assistance with course concepts, and they draw on the collective knowledge of the group by interacting while problem-solving. SI Leaders are students who have previously been successful in the course, and ideally have good communication skills and are motivated to help others become successful. However, unlike a traditional tutoring or recitation model, SI Leaders do not simply give additional instruction to attending students. Instead, they create active lesson plans that foster interactive cognitive input from the group of students in attendance. In this way, students attending the sessions participate in constructing their knowledge, while the SI Leader serves as a guide along the way (Blanc et al., 1983; Congos & Schoeps, 1993). Rather than target high-risk students, SI programs are usually attached to high-risk courses, which often include first year or “gateway” STEM courses. SI is supported by large amounts of evidence that indicate that it is an effective method for improving student outcomes in these courses (Blanc et al., 1983; Congos & Schoeps, 1993; Hensen & Shelley, 2003; Martin & Arendale, 1992).

### The impact of SI on students identifying with different racial/ethnic groups

Students identifying with HU groups often enter college at an academic disadvantage due to coming from inequitable K-12 school systems compared to their peers, and thus face additional challenges in college (President’s Council of Advisors on Science & Technology, 2012). In some studies, SI and similar peer-assisted study sessions have been shown to particularly benefit students who enter college at an academic disadvantage. For example, Dancer et al. examined the effect of peer-assisted study sessions in a first-year business statistics course and found that lower-achieving and international students received the largest benefit from attending sessions (Dancer et al., 2015). Yue et al. looked at data from 44 SI-supported courses and studied the impact of SI on students in different ‘disadvantaged’ groups. They found that students who required English or Math remediation upon matriculation saw larger gains from frequent SI attendance compared to students who did not require remediation (Yue et al., 2018). As noted above, our previous work revealed that GGC’S PSI program particularly benefitted academically disadvantaged students (those who entered GGC with lower HS GPAs) (Achat-Mendes et al., 2020).

In addition to these studies on the impact of SI on students entering college with different levels of academic advantage/preparedness, the impact of SI on students identifying with HU groups has also been previously investigated. Some reports have indicated that SI and similar peer-led academic support models particularly benefit students identifying with HU groups in their STEM courses (Bowman et al., 2021; Peterfreund et al., 2008; Preszler, 2009; Rabitoy et al., 2015; Rath et al., 2007; Yue et al., 2018). Other studies have seen no significant differences between students identifying with HU vs. non-HU groups (Rath et al., 2012). However, none of these studies have fully investigated the impact of SI on students in STEM courses identifying with different racial/ethnic groups while simultaneously accounting for prior academic experiences/preparedness, despite the fact that students identifying with different racial/ethnic groups often have disparate K-12 experiences.

Peterfreund et al. (2008) examined the impact of SI in multiple introductory STEM courses and concluded that students identifying with HU groups saw a larger benefit from attending SI sessions. However, based on the reported grade data, this was only the case for courses in which students identifying with HU groups had lower course grades than students identifying with non-HU groups. When examining courses where students from both groups had similar course performance, all students benefitted equally from SI sessions. No attempt was made to correct for prior academic performance, so the effect of students’ prior academic experiences is unclear. Similarly, Preszler (2009) reported that replacing lecture with peer-led workshops led to larger increases in AB rates and reductions in DFW rates for students identifying with HU groups. However, these students were underperforming in the course compared to their peers identifying with non-HU groups, and again, no attempt was made to correct for prior academic performance.

Yue et al. (2018) investigated SI’s impact on students identifying with or belonging to different ‘disadvantaged’ groups. They saw that students identifying with HU groups received a disproportionate benefit from attending SI compared to students identifying with non-HU groups. However, students identifying with HU groups also had lower average course grades compared to their peers, and no attempt was made to correct for prior academic preparedness. Yue et al. did also consider the effect of SI on students who did and did not require English and/or Math remediation, had First Generation Status, or were Pell Eligible, and even looked at the combined impact of multiple ‘disadvantage’ factors. They found that students with multiple disadvantage factors had lower average course GPAs, but also had the largest benefit from SI attendance. However, they did not disaggregate the data, and for example compare the performance of students identifying with HU vs. non-HU groups who did or did not require remediation. Therefore, it is again difficult to conclude if the particular benefit of SI to students identifying with HU groups was related to academic preparedness or to other factors.

Rabitoy et al. (2015) performed a multiple regression analysis on the impact of SI and reported a larger effect size of SI participation for students identifying with HU groups. Although prior GPA and scores on math and English placement exams were utilized in their multiple regression model, the actual prior GPAs, placement scores, and final SI course GPAs were not provided in the report. It is therefore unclear if students identifying with HU and non-HU racial/ethnic groups experienced different levels of prior academic preparedness, and how differently they performed in the courses under study, both with and without SI.

A recent study by Bowman et al. (2021) examined the impact of SI in 21 courses across two semesters, and reported greater gains for students identifying with HU groups. However, the prior or current academic performance of students identifying with HU vs. non-HU groups was not compared or controlled for, so the effect of academic preparedness is unclear. They also did not account for frequency of attendance when comparing students identifying with HU vs. non-HU groups, only whether or not students did or not attend SI. Since they were examining results at a predominantly White institution, they attributed the additional benefit of SI to students identifying with HU groups to an increased sense of community and belonging at the college. Interestingly, Bowman et al. (2021) also examined the effect of SI on all students with different pre-college preparedness levels (as determined by HS GPA and standardized test scores) and found no differences, contrary to the results seen in other studies (Achat-Mendes et al., 2020; Dancer et al., 2015; Yue et al., 2018).

Overall, an examination of these previous studies reveals that the benefit of structured peer-led study sessions to students identifying with HU groups may be closely tied to their academic preparedness. Additional studies investigating the dual impact of both academic preparedness and students’ self-identified race/ethnicity at institutions of varying types and demographics are thus needed.

### Contributions of current study

In this study, we build on our previous work by investigating the impact of PSI participation on students identifying with HU racial/ethnic groups in several introductory STEM courses, while controlling for HS GPA and frequency of PSI attendance. In doing so, we seek to fill an important gap in the literature by investigating the impact of an SI program on students identifying with HU groups, while simultaneously exploring the intersectionality of academic preparedness/prior academic experiences and race/ethnicity. Additionally, we investigated these factors as a function of increasing PSI participation, rather than using PSI participation as a binary variable (i.e., did or did not attend), a common shortcoming in SI analyses (Dawson et al., 2014; McCarthy et al., 1997; Yue et al., 2018). Assessing the impact of PSI as a function of increasing PSI participation can help determine if students must participate in a certain number of sessions to see a significant benefit, and if attending more than this “threshold” number of sessions results in additional gains in student performance.

### Institutional context

**Fig. 1:**
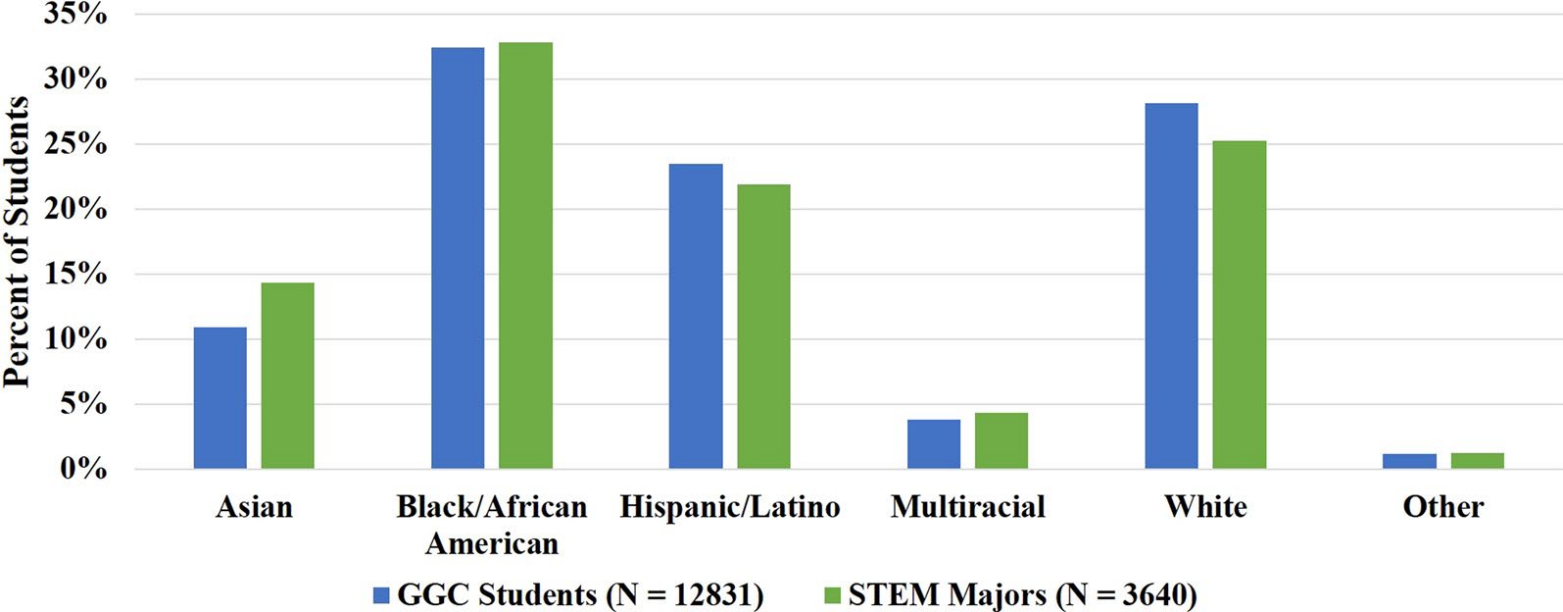
Self-identified racial and ethnic demographic distributions of all GGC students (*N* = 12,831) compared to students enrolled in STEM majors at GGC (*N* = 3640) in Fall 2019

The School of Science and Technology at GGC is the largest school at the college, with approximately 3600 students in Fall 2019. Students identifying with HU racial and ethnic groups are well-represented in STEM at GGC, as shown in Fig. 1,^1^; approximately 60% of STEM students identify with a HU racial/ethnic group. However, these students enter GGC with lower average HS GPAs compared to students identifying with non-HU groups. This is shown in Table 1, which gives the average HS GPA and DFW rates for 7 gateway STEM courses for students identifying with various racial/ethnic groups at GGC, averaged over Fall and Spring semesters from Spring 2017–Spring 2019. HS GPA is not used here as a measure of academic ability, but instead is reflective of prior academic experiences. The data in Table 1 suggest that the prior academic experiences of students identifying with HU vs. non-HU groups may have been unequal, and therefore that these groups of students entered college at different levels of academic advantage/disadvantage. Since HS GPA has been shown to be a strong predictor of later college success (Allensworth & Clark, 2020), this also indicates that students identifying with HU groups at GGC may have greater difficulty transitioning to the college environment. This is reflected in the average DFW rates for so-called “gateway” STEM courses at GGC (General Biology I & II, General Chemistry I & II, College Algebra, Precalculus, and/or Programming Fundamentals), also shown in Table 1. Although STEM courses at GGC heavily employ active learning and other practices shown to improve student outcomes, students identifying with HU groups consistently receive Ds, Fs, and Ws in these gateway courses at higher rates compared to students identifying with non-HU groups (Table 1). Research indicates that student performance in early college coursework heavily impacts student retention and progression (Adelman, 1999). On the other hand, some studies have indicated that once students identifying with HU groups do succeed in their gateway courses, they become more persistent than students identifying with non-HU groups (Alexander et al., 2009; Harris et al., 2020). Supporting students identifying with HU groups in their gateway STEM courses is thus an important component of improving retention and graduation rates for these students.

**Table 1:** Mean high school GPA (HS GPA) and DFW rates disaggregated by students’ self-identified race/ethnicity

	Asian	Black/African American	Hispanic/Latino	Multiracial	White	Other
Mean HS GPA (SEM)	3.07 (0.01)	2.77 (0.01)	2.96 (0.01)	2.94 (0.02)	3.13 (0.01)	2.91 (0.04)
*DFW rates*						
General Biology I	27.3%	40.3%	30.0%	35.3%	21.4%	25.0%
General Biology II	20.7%	29.1%	22.3%	31.8%	16.8%	37.5%
General Chemistry I	30.0%	52.7%	41.0%	43.6%	31.3%	57.1%
General Chemistry II	25.2%	41.3%	34.5%	38.1%	27.5%	15.0%
College Algebra	27.5%	45.8%	35.3%	38.5%	30.4%	41.8%
Precalculus	27.3%	52.2%	36.3%	41.6%	28.0%	51.3%
Programming Fundamentals	33.3%	55.8%	44.4%	43.6%	35.2%	28.6%

For HS GPAs, numbers in parentheses indicate standard errors of the means (SEM)

One method of supporting students in these courses at GGC is through our Peer Supplemental Instruction (PSI) program. As noted above, we previously reported that frequent PSI participation was correlated with improved course outcomes, and the program appeared to be particularly beneficial for academically disadvantaged students, as determined by their incoming high school grade point average (HS GPA). It therefore seems probable that students identifying with HU groups at GGC may derive a larger benefit from PSI compared to their peers identifying with non-HU groups. If analysis reveals that to be the case, it is important to determine if this effect is solely due to differences in pre-college academic advantages/disadvantages, or if PSI offers additional assistance to students identifying with HU groups at GGC even after controlling for academic preparedness/prior academic experiences.

### Peer supplemental instruction at GGC

Typical of many SI programs, the PSI program at GGC utilizes student PSI Leaders who previously did well in the course. PSI Leaders plan and facilitate regular PSI sessions outside of normal class hours which students currently enrolled in the course may attend. However, this program has several modifications compared to a traditional SI model that are designed to accommodate the small class sizes and high section counts of GGC classes.

In the traditional SI model, leaders facilitate sessions for a class of students taught by a single instructor. In contrast, PSI Leaders prepare collaborative activities and lead sessions for students across multiple sections of the same course that are taught by different instructors and that may be on different schedules. Since students in a single session come from multiple sections of the course with various professors, they may be working on different material which has been taught to them in a variety of ways. Our undergraduate PSI Leaders are adaptable and skilled in managing multiple groups of students working on different topics in one PSI session. Along with review of course material, PSI sessions also provide an emphasis on metacognitive skills and incorporate STEM skills in practice.

Typically, 1–3 PSI Leaders are assigned to a specific course, depending on the number of sections of the course offered that semester; this may vary from ~ 10 sections to over 50 sections, depending on the subject. PSI Leaders offer 3–6 PSI sessions (55 min each) for that course at varying times throughout the week. Students enrolled in the course may attend any or all of the offered PSI sessions. The PSI sessions are offered at the same times each week and the times are determined by the availabilities of the PSI leaders and students taking the course. In many cases, PSI leaders use surveys at the start of the semester to find times that work best for the students. Session times are advertised by instructors teaching the course, flyers and digital signage on campus, and direct emails to students enrolled in PSI-supported classes. A website is updated every week with the session times (although these typically do not change) and the topics that will be covered in each section. Like most SI programs, student attendance is voluntary, resulting in a significant degree of variability in student participation. In light of this, the effectiveness of PSI on student course performance was measured as a function of attendance in previous and current studies of our program. The number of students that attend each session can vary significantly based on factors such as the specific course, the time and day of the session, the individual PSI leader, and class specific factors (like an upcoming exam). Approximately 10% of students enrolled in a PSI-supported course attend one or more PSI sessions, and individual PSI sessions typically see attendance numbers between 2 and 15 students. The PSI program at GGC is currently managed and coordinated by a team of STEM faculty who interview, hire, and train PSI Leaders, and then mentor them throughout the semester through weekly meetings that focus on lesson planning, PSI session strategies, academic mindset, and professional development. In many other SI implementations, administrative responsibility for the program is placed under the division of academic or student affairs. The elements, design, and effectiveness of the PSI model at GGC were previously described in detail, with evidence of the model’s success in improving participant course grades, attitudes toward STEM coursework, and career competencies in PSI Leaders (Achat-Mendes et al., 2020).

### Research questions

SI appears to particularly benefit students who are underserved and/or underprepared for college, as well as students identifying with HU racial and ethnic groups. However, no study has fully investigated the impact of SI on students while accounting for both of these variables. Our research seeks to understand how the PSI program at GGC impacts students identifying with HU vs. non-HU racial/ethnic groups, while simultaneously accounting for prior academic experiences. In order to characterize the intersection of these variables, the study investigated the following specific questions:

1. To what extent does the frequency of PSI participation affect the final course grades of participants who identify with HU racial/ethnic groups versus those who identify with non-HU racial/ethnic groups?
2. To what extent does the effect of PSI on students identifying with different racial/ethnic groups depend on their prior academic experiences?

## Methods

### Data collection and analysis

Final course grades and demographic data (identified race/ethnicity, high school GPA (when available), and course GPA) were collected for all PSI participants at the conclusion of each semester. Data were collected for 7 PSI-supported courses (General Biology I and II, General Chemistry I and II, College Algebra, Precalculus, and Programming Fundamentals) each Fall and Spring semester from Spring 2017–Spring 2019 (5 semesters total). Fall 2018 data also include Organic Chemistry I, and Spring 2019 data also include Organic Chemistry I and Cell Biology. All data collection protocols were reviewed and approved by GGC’s Institutional Review Board.

Data were aggregated across all courses to analyze the possible impact of PSI participation on final course grades. Students were often enrolled in multiple PSI-supported courses at one time, or for sequential semesters. If students attended PSI for multiple courses in the data collection period, their data would be reported multiple times here (once per PSI-supported course). Analyses were conducted by first grouping participants into one of four categories of attendance (1–2, 3–5, 6–9, or 10 + PSI sessions) for a specific PSI course, and then analyzing the association between attendance and final course grade and/or final course GPA. Final course GPAs were assigned numerical values as follows: *A* = 4, *B* = 3, *C* = 2, *D* = 1, and *F*/*W* = 0.^2^

To evaluate any differential effects of PSI participation on student performance, these analyses were repeated after disaggregating students into the five racial/ethnic groups in which they institutionally self-identified. The categories are: Asian, Black/African American, Hispanic/Latino, White, and Other. The Other category includes students identifying as American Indian, Alaskan Native, Multiracial, Native Hawaiian, Pacific Islander, and Race Unknown. These groups were combined for the purposes of this study due to small sample sizes. For all analyses, data from individual students were averaged to obtain group means.

Statistical methods that were implemented to determine whether there was any statistical significance of participation in PSI included a one-way or two-way analysis of variance (ANOVA). The testing of significant main effects was followed by pairwise comparisons, using Tukey *t*-tests. In addition, two moderator tests were conducted. The moderation effect was examined by making our dependent variable (GPA) the product of levels of the independent variable (PSI attendance) and our moderator variables (self-identified race/ethnicity, and high school GPA) were run. The criterion for significance was *p* < 0.05 for all analyses conducted in this study.

## Results and discussion

### Profile of PSI attendees

**Fig. 2:**
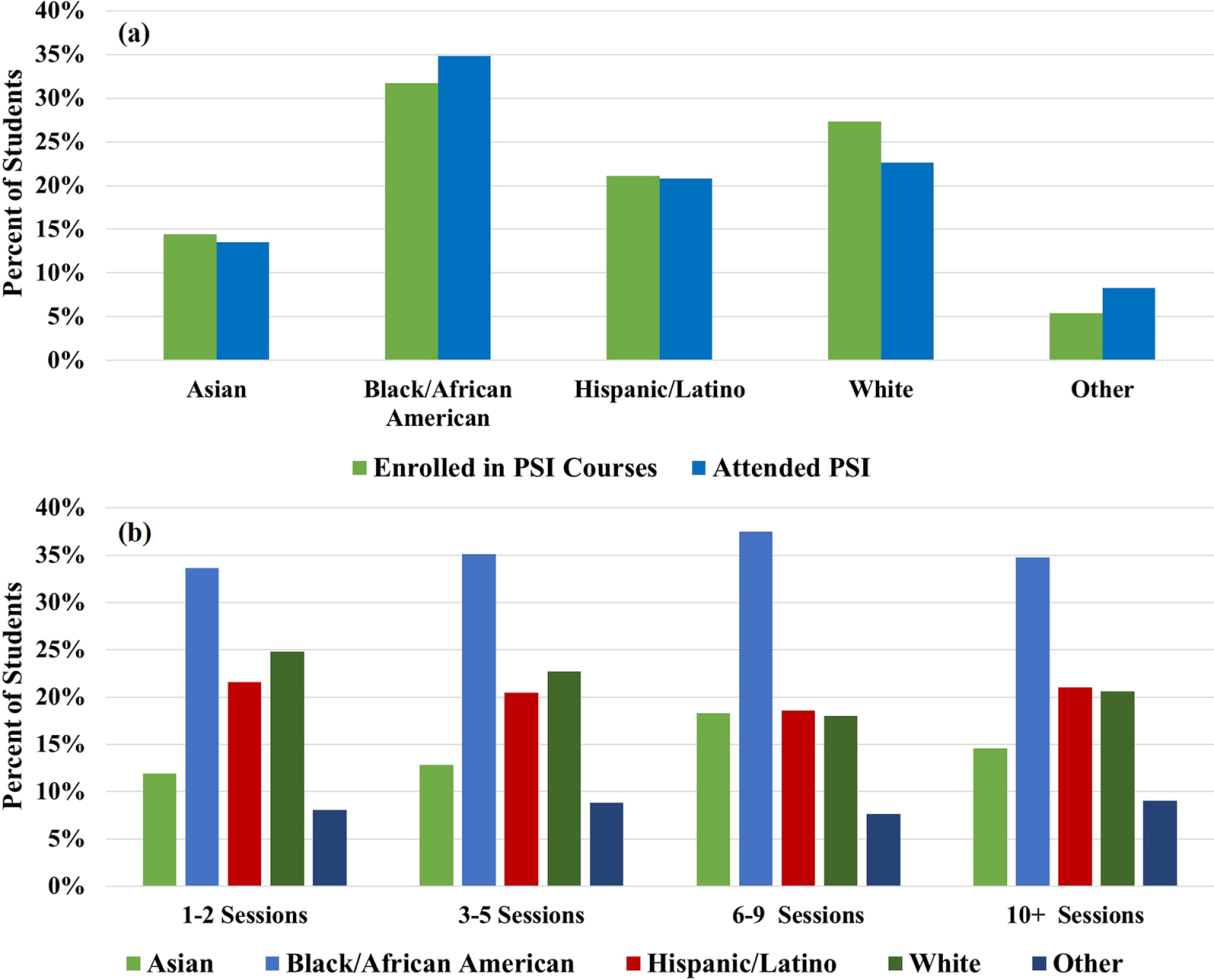
**a** Self-identified racial/ethnic demographic distribution of students enrolled in PSI-supported courses vs. students attending PSI sessions. **b** Self-identified racial/ethnic demographic distribution of students attending PSI sessions, separated by frequency of attendance (1–2, 3–5, 6–9, or 10 + PSI sessions for a given course within a single semester)

*Racial/ethnic demographics*. Figure 2a shows the self-identified racial and ethnic demographic distribution of students who attended at least one PSI session for a PSI-supported course in a given semester, compared to the demographics of all students enrolled in PSI-supported courses. PSI attendees strongly resembled the overall population of students enrolled in PSI-supported courses. However, PSI attendees were slightly more likely to identify as Black/African American or as one of the racial/ethnic groups in the Other category, and slightly less likely to identify as White or Asian, compared to the overall student population. This is also reflected in Table 2, which shows the percentage of students who were enrolled in a PSI-supported course and attended at least one PSI session, separated according to students’ self-identified race/ethnicity. The fact that students identifying with HU groups were slightly more likely to attend PSI is in agreement with other reports (Kudish et al., 2016; McGee, 2005; Moore & LeDee, 2006; Peterfreund et al., 2008). The racial/ethnic demographic distribution for PSI attendees remained very similar when analyzed according to frequency of attendance (Fig. 2b), indicating that frequency of attendance was similar for students identifying with all races/ethnicities.

**Table 2:** PSI participation as a percent of students enrolled in PSI-supported courses, disaggregated by students’ self-identified race/ethnicity

	All students	Students identifying as:				
		Asian	Black/African American	Hispanic/ Latino	White	Other
Percent of population	10.0%	9.4%	10.9%	9.8%	8.3%	15.3%

*HS GPA.* Table 3 shows the mean HS GPAs for PSI attendees and for all students enrolled in PSI-supported courses, separated by students’ self-identified race/ethnicity. For students who attended PSI sessions, the mean HS GPAs in Table 3 are also broken down as a function of PSI attendance (1–2, 3–5, 6–9, or 10 + sessions). Again, it is important to note that HS GPA is not necessarily being used here as an indicator of student motivation or ability, but instead may be reflective of the different high school environments (funding, teacher/student ratio, available coursework, etc.) experienced by each student, and thus reflects how prepared they were to transition to the college environment. HS GPA is also commonly used as a tool to identify self-selection bias for academic support services, i.e., the idea that only strong students attend SI services (Dawson et al., 2014).

**Table 3:** Mean high school GPAs for PSI attendees (disaggregated by PSI attendance) and for all students enrolled in PSI-supported courses, disaggregated by students’ self-identified race/ethnicity

	Average high school GPA (SEM)				
	1–2	3–5	6–9	10 +	All students
Asian	3.07 (0.07)	3.08 (0.08)	3.26 (0.08)	3.15 (0.11)	3.08 (0.01)
Black/African American	2.74 (0.03)	2.86 (0.05)	2.86 (0.05)	2.61 (0.06)	2.78 (0.01)
Hispanic/Latino	2.92 (0.04)	3.11 (0.07)	3.11 (0.07)	3.14 (0.08)	2.96 (0.01)
White	3.17 (0.04)	3.27 (0.05)	3.17 (0.07)	3.20 (0.08)	3.14 (0.01)
Other	2.94 (0.07)	3.12 (0.09)	2.79 (0.12)	2.94 (0.05)	2.93 (0.02)

Numbers in parentheses are standard errors of the means (SEM)

A one-way ANOVA revealed significant effects of race/ethnicity, *F* (4, 1605) = 37.48, *p* < 0.0001, on mean HS GPA. Students identifying as Black/African American, Hispanic/Latino, and with one of the races/ethnicities in the Other category had significantly lower mean HS GPAs compared to their peers who identified as White or Asian. This indicates that these students were at the greatest disadvantage when entering college. It is interesting to note that while students identifying with HU groups had the lowest average HS GPAs, they also had the highest rates of PSI attendance (Fig. 2). It is encouraging that the students most in need of academic support were also the most likely to seek it out.

Statistical analysis showed no correlation between HS GPA and PSI attendance for students identifying as Asian, Black/African American, White, or a race/ethnicity in the Other category. Furthermore, no significant difference existed between the mean HS GPA of PSI attendees vs. the overall average for all students enrolled in these courses within each racial/ethnic group. These results suggest that for students identifying with these racial/ethnic groups, college preparedness was not a good predictor of who would attend PSI, or if they did attend, who would attend more frequently. Any differences in final course grades can thus be attributed (at least to some extent) to the effect of attending PSI sessions.

On the other hand, a significant correlation between HS GPA and PSI attendance did exist for students identifying as Hispanic/Latino (*r*(349) = 0.16, *p* < 0.01); students identifying as Hispanic/Latino who attended PSI 3 + times per semester had slightly higher (0.15–0.18) mean HS GPAs compared to that of all students identifying as Hispanic/Latino who were enrolled in PSI-supported courses. Based on this, it appears that students identifying as Hispanic/Latino who attended PSI at least 3 times may have been slightly better prepared for college coursework compared to those who attended infrequently or not at all. This indicates that self-selection bias may have been a factor for these students attending PSI. This point will be discussed further in the next section.

### Final course grades as a function of PSI attendance and self-identified race/ethnicity

**Fig. 3:**
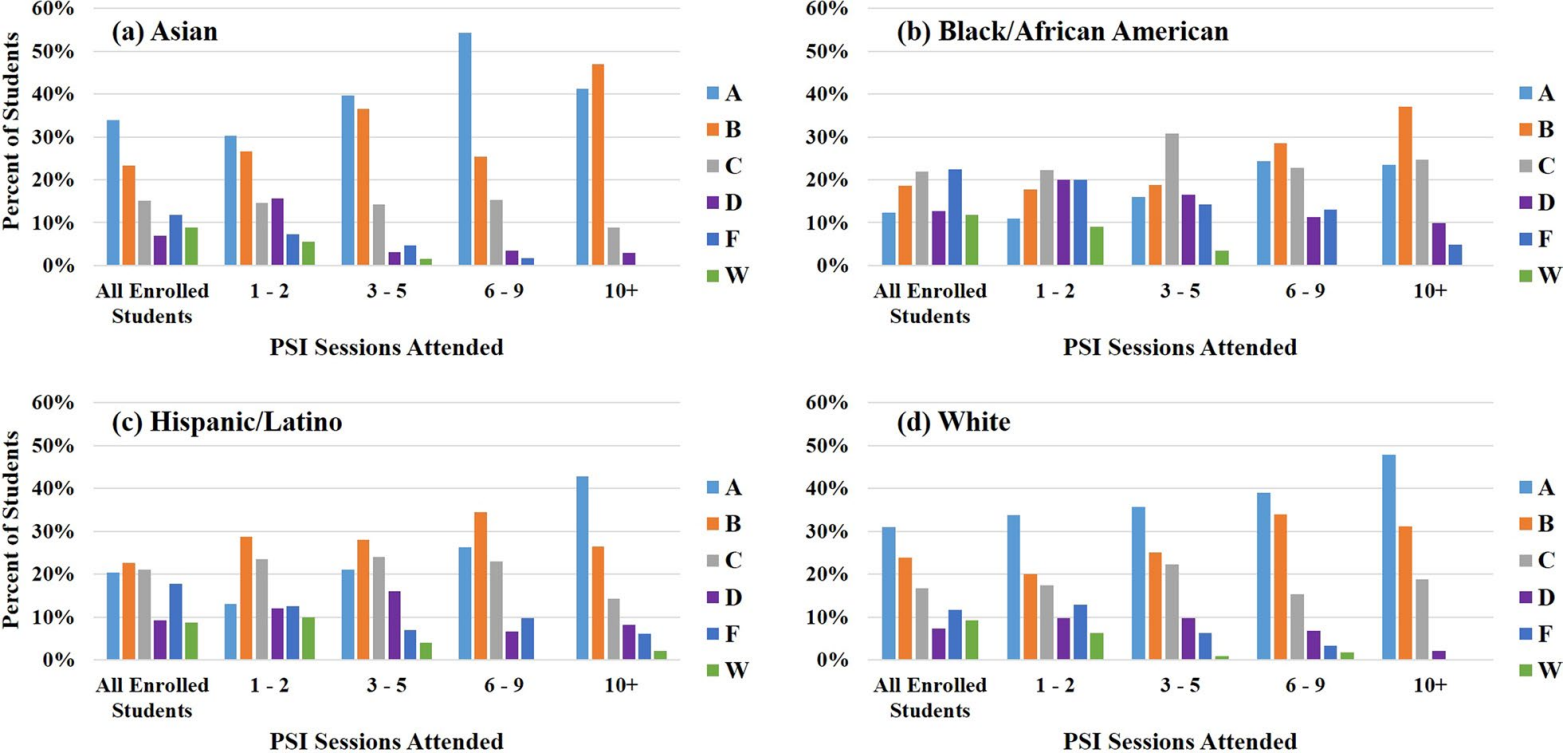
Final course grade distributions of PSI participants, grouped by session attendance, for students identifying as **a** Asian (*N* = 265), **b** Black/African American (*N* = 679), **c** Hispanic/Latino (*N* = 401), and **d** White (*N* = 444). Shown for comparison are the final grade distributions for all students enrolled in PSI courses for students identifying as **a** Asian (*N* = 2834), **b** Black/African American (*N* = 6238), **c** Hispanic/Latino (*N* = 4143), and **d** White (*N* = 5375)

**Fig. 4:**
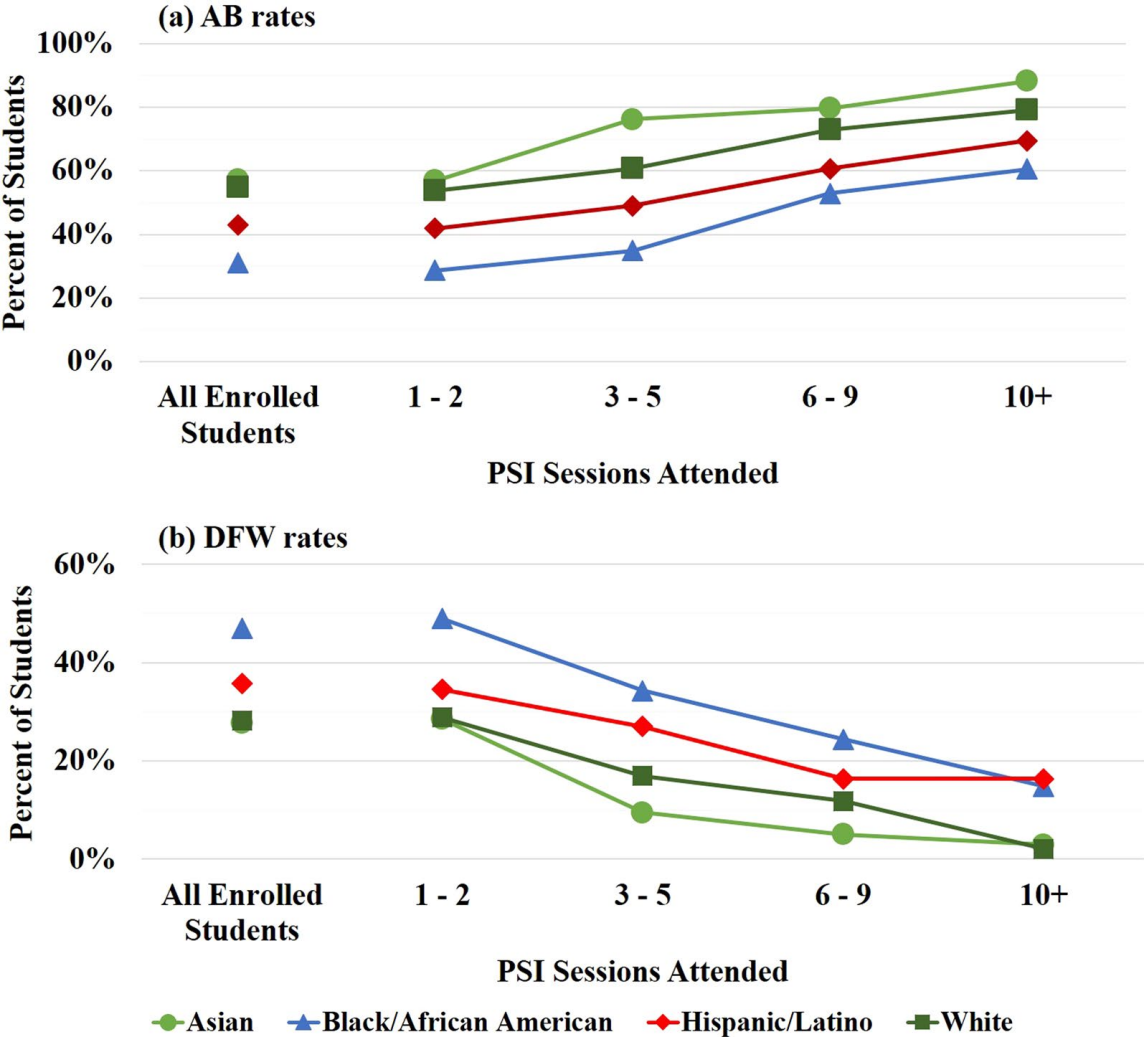
Aggregated **a** AB and **b** DFW rates for PSI attendees as a function of PSI attendance and students’ identified race/ethnicity

Final course grades were examined for PSI attendees as a function of both PSI attendance and students’ identified race/ethnicity; these results are shown in Fig. 3a–d. Also shown for comparison are the final course grades for all students^3^ identifying with each racial/ethnic group who were enrolled in PSI-supported courses. The “Other” category was omitted in this and subsequent analyses due to an insufficiently large sample size for this group, and because this group contained students identifying with a variety of racial/ethnic backgrounds. Students attending 1–2 PSI sessions in a given semester exhibited very similar final course grade distributions and DFW rates compared to those of all enrolled students, regardless of students’ self-identified race/ethnicity. PSI attendees saw increasing AB rates and decreasing DFW rates with increasing PSI attendance; these trends are more clearly seen in Fig. 4a and b. Averaging the data in Fig. 4a and b across all students, there is an average increase in AB rates of 29.0 percentage points, and an average decrease in DFW rates of 26.1 percentage points, for students who attended 10 + vs. 1–2 PSI sessions.

While all students saw improved grades with increasing PSI attendance, marked differences in AB and DFW rates as a function of students’ self-identified race/ethnicity can be seen in Fig. 4a and b. Students identifying as Asian consistently had the highest AB rates, followed by students identifying as White, Hispanic/Latino, and Black/African American, respectively. The reverse trend is seen for DFW rates, except for students attending 10 + sessions. For this group, the DFW rates of students identifying as Asian and White converged (2.9% and 2.1%, respectively), as did the DFW rates for students identifying as Black/African American and Hispanic/Latino (14.8% and 16.3%, respectively). A significant gap exists in the DFW rates of students identifying with HU vs. non-HU groups. Notably, students identifying as Black/African American typically had the lowest AB rates and highest DFW rates. However, when comparing students who attended 1–2 PSI sessions vs. 10 + sessions, this group showed the largest increase in AB rates (from 28.7 to 60.5%) and the largest decrease in DFW rates (from 47.1 to 14.8%). Statistical analysis of the impact of PSI participation on students identifying with different racial/ethnic groups is discussed in more detail below.

**Fig. 5:**
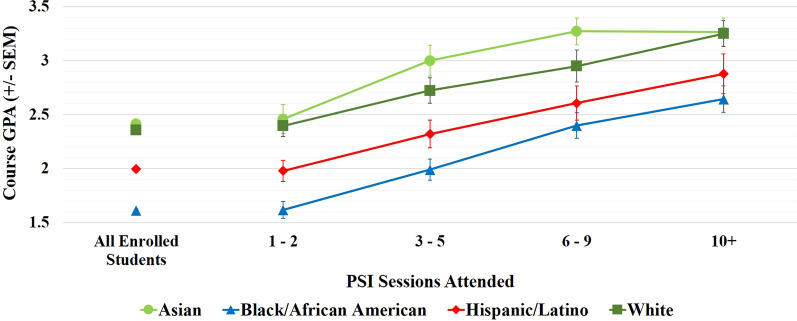
Mean final course GPAs of PSI participants (*N* = 1789) as a function of PSI attendance, and of all students enrolled in PSI-supported courses (*N* = 18,590), for students identifying with the four racial/ethnic groups studied here. Points are aggregate means ± SEM of student final course GPAs from nine foundation courses over 5 semesters

Mean final course GPA was also investigated as an overall indicator of successful course completion and used for further statistical analysis. Figure 5 shows the mean final course GPA for PSI attendees as a function of both students’ self-identified race/ethnicity and number of PSI sessions attended. A two-way ANOVA revealed significant effects of both students’ self-identified race/ethnicity (*F* (3, 1773) = 30.18, *p* < 0.0001) and PSI attendance (*F* (3, 1773) = 35.25, *p* < 0.0001) on final course GPA, though no interaction was found between these two factors. This indicates that attending PSI sessions had a similar effect on overall course GPA for students identifying with all racial/ethnic groups. This was confirmed using a moderator test, which also showed that there was no significant interaction between PSI attendance and students’ self-identified race/ethnicity on course GPA (*b* = 0.006, SE = 0.023, *p* = 0.794, CI = (− 0.039, 0.051)). Thus, the effect of attendance on GPA does not depend on students’ self-identified race/ethnicity (i.e., there was no significant moderation effect). However, the moderator test found that the main effect of attendance was significant as it influenced the course GPA (*b* = 0.300, SE = 0.067, *p* < 0.001, CI = (0.170, 0.430)), confirming the results from the two-way ANOVA.

Analyzed separately via one-way ANOVAs, students identifying with the four racial/ethnic groups analyzed here all showed a statistically significant increase in final course GPA with increasing PSI attendance. Although there was no statistical difference in the overall effect of attending PSI for students identifying with different racial/ethnic groups in the two-way ANOVA, comparing the results of the one-way ANOVAs, the effect size was largest for students identifying as Black/African American (*F* (3, 675) = 18.79, *p* < 0.0001). These students saw an average increase of 1.03 GPA units (i.e., one letter grade) for students attending 10+ vs. 1–2 sessions. Students identifying as Asian (increase of 0.81 GPA units, *F* (3, 261) = 8.26, *p* < 0.0001), Hispanic/Latino (increase of 0.90 GPA units, *F* (3, 397) = 8.04, *p* < 0.0001), and White (increase of 0.85 GPA units, *F* (3, 440) = 7.02, *p* < 0.0001) all had similar gains to each other.

Further analysis also revealed differences in the threshold level of PSI required to see a significant improvement in final course GPA. Tukey post hoc analyses showed that while students identifying as Black/African American and Asian exhibited significant increases in course GPA when attending just 3–5 sessions (*p* < 0.05), students identifying as Hispanic/Latino and White required at least 6+ sessions to see a significant effect (*p* < 0.05). At the same time, students identifying as Asian appeared to experience a ceiling effect, seeing no additional benefit from participating in 10 + sessions as compared to 6–9 sessions. Students identifying with all other racial/ethnic groups continued to see increasing benefit with increasing PSI attendance. Overall, as students identifying as Black/African American saw improved final course grades from attending as few as 3–5 PSI sessions, continued to see an increasing benefit with increasing PSI attendance, and exhibited the largest overall increase in their final course grade when attending 10+ vs. 1–2 sessions, they appear to have gained the largest benefits from participating in PSI.

As noted above, the two-way ANOVA also revealed significant effects of students’ self-identified race/ethnicity on final course GPA. In other words, students identifying with different races/ethnicities had significantly different final course GPAs. Students identifying as Black/African American had the lowest overall course GPA (mean course GPA of 1.98 across all attendance groups), followed by students identifying as Hispanic/Latino. Students identifying as Asian consistently had the highest overall course GPA, with a mean course GPA of 2.88 across all attendance groups. These results are consistent with the trends in AB rates and DFW rates seen in Fig. 4, and are seen across all attendance levels; even with frequent PSI attendance (10+ sessions/semester), students identifying as Black/African American and Hispanic/Latino had lower final course GPAs compared to their peers who identified as White and Asian.

One interesting point is that although students identifying as Hispanic/Latino who attended 3 + PSI sessions had similar HS GPAs compared to students identifying as White and Asian (Table 3), they received lower grades and final course GPAs for all levels of PSI attendance (Figs. 3, 4, 5). In fact, while the performance of students identifying as White and Asian essentially converged when attending 10 + PSI sessions, students identifying as Hispanic/Latino in this attendance group instead performed more similarly to students identifying as Black/African American, despite being seemingly better prepared. This removes some concern regarding self-selection bias for PSI attendees identifying as Hispanic/Latino, as self-selection bias should result in higher final course grades than expected, not lower. Moreover, students identifying as Hispanic/Latino who attended 10 + sessions per semester had better course outcomes compared to students who only attended 3–5 sessions, although they had similar HS GPAs. Together this suggests that although PSI attendees identifying as Hispanic/Latino may have been slightly better prepared for college coursework in general (compared to students identifying as Hispanic/Latino who did not attend PSI), they still directly benefitted from attending PSI sessions. Indeed, the fact that they underperformed compared to PSI attendees identifying as White and Asian, despite having similar HS GPAs, indicates that there may be additional factors negatively affecting the performance of students identifying with HU groups that are not considered here.

In summary, PSI attendance was positively correlated with improved course outcomes for students identifying with all racial/ethnic groups. The degree of benefit increased with the number of PSI sessions attended. Frequent PSI attendance resulted in drastically reduced DFW rates, decreasing from an average of 35% for students attending 1–2 sessions to 10% for students attending 10 + sessions (Fig. 4b). Average AB rates simultaneously increased from 46 to 75% (Fig. 4a), and overall final course GPAs increased by roughly one letter grade (Fig. 5). At the same time, the consistent disparity in final grades between students identifying with different racial/ethnic groups indicates that while PSI is a promising method to support student success, it is not able to fully compensate for the fact that students identifying with HU groups enter GGC at an academic disadvantage compared to their non-HU peers, and/or experience additional educational barriers once they are in college.

### Interaction between college preparedness, PSI attendance, and race/ethnicity

In our previous work, we demonstrated that PSI is particularly beneficial for underprepared students, as determined by their HS GPA (Achat-Mendes et al., 2020). Since students identifying with different races/ethnicities enter GGC with different average HS GPAs, we first sought to confirm these findings, so that we could further investigate how students identifying with different racial/ethnic groups benefitted from PSI. A two-way ANOVA revealed significant effects of both HS GPA (*F* (2, 1442) = 72.07; *p* < 0.0001) and PSI attendance (*F* (3, 1442) = 18.40; *p* < 0.0001) on final course GPA for PSI attendees. In addition, the ANOVA revealed a significant interaction between these two factors (*F* (6, 1442) = 2.20; *p* < 0.05). The interaction between HS GPA and PSI attendance was confirmed via a second moderator test, in which we examined whether the relationship between the PSI attendance of students and their final course GPA was influenced by their high school GPA (i.e., preparedness). The model showed there was a significant interaction between PSI attendance and HS GPA on final course GPA (*b* = − 0.135, SE = 0.047, *p* = 0.004, CI = (− 0.227, − 0.043)). These analyses indicate that while students with lower HS GPAs received lower final course grades in their PSI-supported STEM courses compared to students with higher HS GPAs, they also received a greater benefit from frequent PSI participation. These trends may in part explain why students identifying as Black/African American, who entered GGC with the lowest average HS GPA, experienced the largest benefit from PSI participation. However, it is also possible that PSI offered additional benefits to students identifying with HU groups related to a sense of belonging and peer mentorship.

**Fig. 6:**
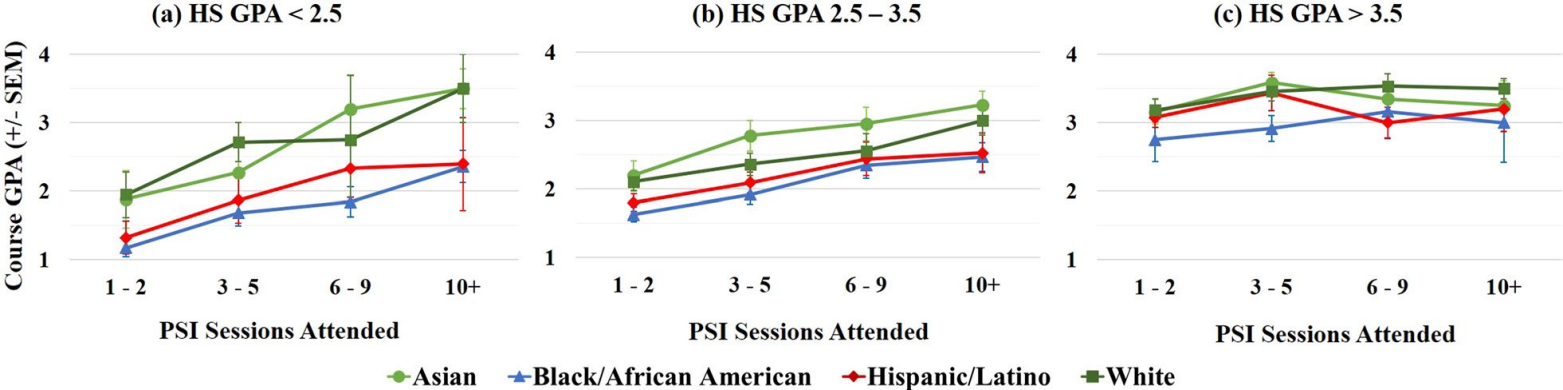
Comparison of the effects of PSI participation on final course GPA for students identifying with the four racial/ethnics group investigated here, separated by incoming HS GPA: **a** < 2.5 (*n* = 307), **b** 2.5–3.5 (*n* = 829), and **c** > 3.5 (*n* = 318)

To investigate this further, the effect of PSI attendance on final course GPA was again analyzed as a function of students’ self-identified race/ethnicity, but this time students were separated into three groups, as shown in Fig. 6: underprepared (HS GPA < 2.5, Fig. 6a), moderately prepared (HS GPA 2.5–3.5, Fig. 6b), and well prepared (HS GPA > 3.5, Fig. 6c) for college-level coursework. Two-way ANOVAs revealed significant effects of both PSI attendance and of race/ethnicity on final course GPA for groups with HS GPA < 2.5 [attendance, *F* (3, 291) = 8.13; *p* < 0.0001 and race/ethnicity, *F* (3, 291) = 6.24; *p* < 0.001] and HS GPA 2.5–3.5 [attendance, *F* (3, 813) = 13.84; *p* < 0.0001 and race/ethnicity, *F* (3, 813) = 7.58; *p* < 0.0001]. No significant effects of either were found for students with HS GPA > 3.5, and no significant interaction was found between race/ethnicity and PSI attendance in the two-way ANOVAs for any HS GPA group.

First, we can examine the overall relationships between HS GPA, PSI attendance, and final course GPA shown in Fig. 6. Averaging the data across students identifying with all racial/ethnic groups, underprepared students saw an average increase of 1.25 GPA units when attending 10 + vs. 1–2 PSI sessions. Moderately prepared students saw an average increase of 0.85 GPA units when attending 10 + vs. 1–2 PSI sessions. Well prepared students did not see an impact even from frequent PSI participation; however, this may simply be because this group already performed very well, with a mean final course GPA of 3.22 across all attendance groups. These results are consistent with previous reports (Achat-Mendes et al., 2020; Dancer et al., 2015; Yue et al., 2018). Thus, the current study adds support to previous research indicating that structured peer-led study methods particularly benefit underprepared students. The fact that the least prepared students saw the largest benefit from PSI also strengthens the conclusion that the improvement in course outcomes was a direct result of attending PSI sessions and not due to PSI attendees being inherently stronger students.

**Fig. 7:**
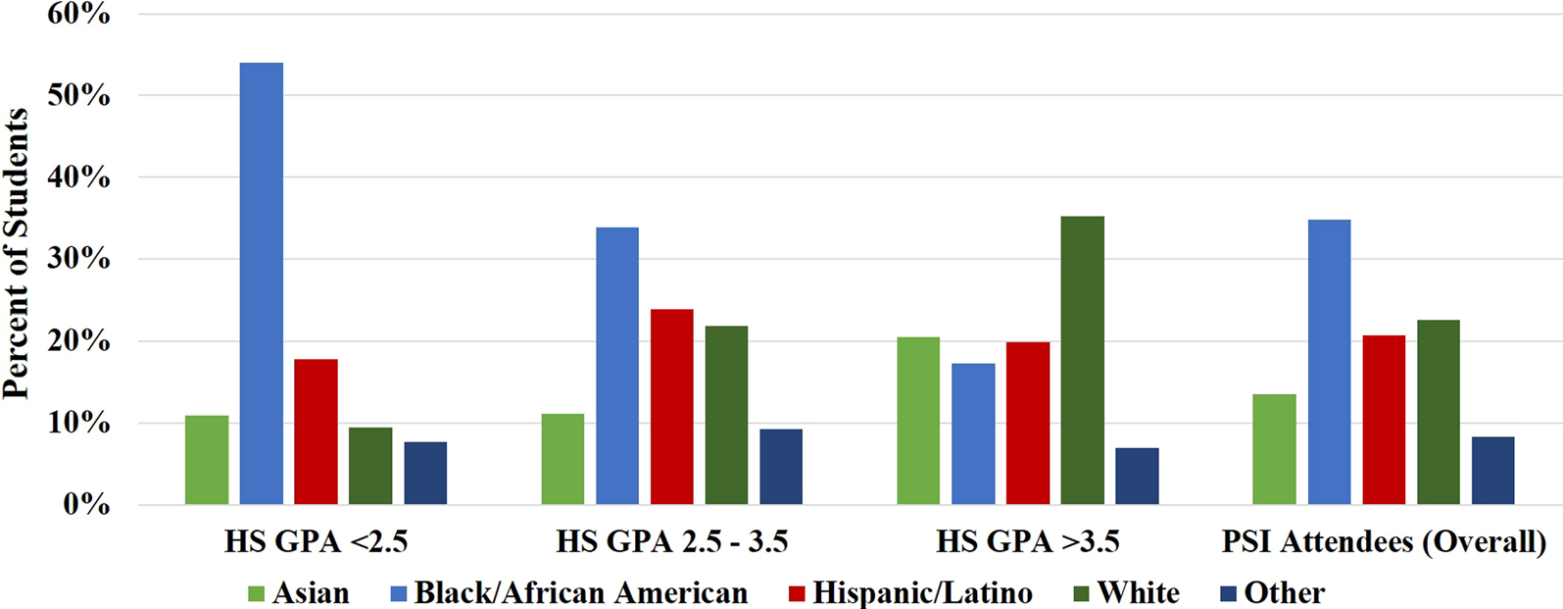
Self-identified racial/ethnic demographic distribution of students attending PSI sessions, separated by HS GPA, and compared to the self-identified racial/ethnic demographic distribution of all PSI attendees

Figure 6 also allows us to more closely examine the relationships between HS GPA, PSI attendance, and final course GPA, this time exploring differences between students identifying with different racial/ethnic groups. For underprepared and moderately prepared students, students identifying with HU groups had significantly lower mean course GPAs compared to their similarly prepared peers for all levels of PSI attendance, as shown in Fig. 6a and b. Within each HS GPA group, students identifying with all racial/ethnic groups experienced a similar increase in their final course GPA with increasing PSI attendance. The result is that there was a consistent gap in the mean final course GPAs for under- and moderately prepared students identifying with HU vs. non-HU groups. This gap was largest for the underprepared students (Fig. 6a), and did not diminish even with high levels of PSI participation. Promisingly, there were no significant differences in final course GPAs for well prepared students identifying with different races/ethnicities (Fig. 6c). This was also the only group for which PSI participation did not have a significant impact on final course GPA for any group. This shows that if students enter college well prepared, they will perform equally well, regardless of students’ self-identified race/ethnicity. Unfortunately, underprepared students were much more likely to identify with HU groups, and in particular as Black/African American, compared to moderately or well prepared students, as seen in Fig. 7. This again indicates that students identifying with HU groups were more likely to enter college at an academic disadvantage. As a whole, these results are concerning, as they indicate that academic inequities at the K-12 level resulted in even greater disparities in student success rates at the college level for underprepared and moderately prepared students. Since students identifying with HU groups were more likely to experience these educational inequities, they were also struggling more at the college level.

### PSI’s impact on students identifying with HU groups

Overall, it does not appear that the PSI program at GGC particularly benefitted any students based on their self-identified race/ethnicity alone. Instead, our data extend upon our previous work which showed that PSI addressed differences in college preparedness based on prior academic experiences. The result is that GGC students identifying with some racial/ethnic groups—in particular, students identifying as Black/African American—received a larger benefit from PSI because they arrived at GGC underserved and at an academic disadvantage compared to their peers. These results are important given the previous disagreement in the literature on whether SI is particularly beneficial for students identifying with HU racial/ethnic groups. Additional studies investigating the dual impact of both students’ prior academic experiences and students’ self-identified race/ethnicity at institutions of varying types and demographics may shed additional light on this. For example, future studies might perform a similar investigation at a predominantly white institution to determine if the student demographic makeup influences these results, or at a research-based institution with large class sizes to determine if class format has an effect. In both of these examples, the social benefits of a peer-led academic support program may be more prominent compared to at GGC, where students regularly interact with students and faculty identifying with HU groups in small classroom settings.

These results also reveal a troubling pattern that students identifying with HU groups appear to have additional barriers in both their high school and college education, as evidenced by the different HS GPAs and college STEM course outcomes for these students. While PSI does not equalize final course outcomes between similarly prepared students identifying with HU and non-HU groups, it is still a promising academic support model. PSI disproportionately benefitted less prepared students, and thus helped to close achievement gaps related to college preparedness/prior academic experience. Since students identifying with HU groups had the highest rate of PSI participation (Fig. 2) and made up a majority of the underprepared and moderately prepared groups (Fig. 7), these were also the students who received the greatest benefit from participating in PSI. We propose that this model can be used in conjunction with additional student success support services to more fully address inequities in educational outcomes for students identifying with different racial/ethnic groups, as explained in more detail below.

### Looking towards equitable outcomes

The data shown here give some interesting insights into the factors affecting student performance in introductory STEM courses at GGC. Our findings confirm that preparedness plays a major role in student success; only well prepared students showed a passing mean course GPA for students identifying with all racial/ethnic groups. PSI attendance had no significant effect on final course GPA for this group, suggesting that these students would have been successful even without this academic support service. This group also showed no achievement gap in final course grades for students identifying as Hispanic/Latino and Black/African American. These results indicate that if students identifying with HU groups have strong academic experiences before they enter GGC, they are just as likely to succeed as their peers. Unfortunately, as shown in Fig. 7, students identifying as Black/African American are not well-represented in this group. This disparity must be addressed at the K-12 level in order to put these students on equal footing with their peers. Until that time, however, it is necessary for us to address this inequity as best we can at the college level.

Further study is needed to identify other factors that might affect college performance for students identifying with HU groups attending GGC, and interventions that might assist them. These interventions may or may not be part of the PSI program. For example, the PSI program has recently incorporated academic mindset interventions into PSI sessions, which address growth mindset, sense of belonging, and value of coursework for PSI attendees. Jordt et al. (2017) found that values affirmation interventions in an introductory biology course reduced disparities in course outcomes between students identifying with different racial/ethnic groups, and Fink et al. (2018) showed that incorporating growth mindset interventions in a general chemistry course essentially eliminated them when controlling for prior academic achievement. Incorporating academic mindset activities into PSI may therefore prove particularly beneficial to students identifying with HU groups. Future analysis of the effectiveness of the PSI program will include determining the dual impact of PSI and academic mindset interventions on underprepared students and students identifying with HU groups.

It is also possible that separate interventions could be combined with PSI to further improve course outcomes for students identifying with HU groups. Previous studies have shown that holistic programs show great promise in reducing achievement gaps in STEM. For example, Toven-Lindsey et al. (2015) reported on the Program for Excellence in Education and Research in the Sciences (PEERS) at the University of California, Los Angeles, which combined academic and career seminars, holistic academic counseling, research seminars, and collaborative-learning workshops. Although PEERS enrollment was not limited to students identifying with HU groups, it was targeted towards students underperforming in STEM, including students identifying with HU groups, students identifying as female, and students from lower socioeconomic backgrounds. PEERS participation significantly increased student success in science and math courses, and PEERS students on average took more science classes and had higher retention in science majors after two years. A 2020 report on the Operation STEM (OpSTEM) program at Cleveland State University described similar results. This program utilized two levels of intervention: SI alone, and a comprehensive version (OpSTEM Scholars) in which students attended a 2-week summer bridge program before their first semester, had mandatory SI in their math classes in their first year, and received advising and research opportunities throughout their undergraduate career. While all students benefitted from SI sessions, students identifying with HU groups saw much greater gains when enrolled in the comprehensive OpSTEM Scholars program. In contrast, students identifying with non-HU groups had similar outcomes whether they participated in SI alone or the comprehensive OpSTEM Scholars Program (Van Sickle et al., 2020). A 2016 study by Lane attempted to explain the efficacy of such comprehensive programs through focus groups and interviews with 50 students enrolled in a Comprehensive STEM Program (CSP) at a large mid-western university. The CSP included a summer bridge program, biweekly advising, peer-led recitation sessions, a first-year seminar, clustered residential assignments, and peer mentoring, among other program components. Lane concluded that the impact of such comprehensive programs results from a combination of four basic components: proactive care, holistic support, community building, and catalysts for STEM identity development (Lane, 2016). At GGC, PSI is part of a larger systems model designed to reform STEM education through the use of HIPs, CUREs, and PSI (Achat-Mendes et al., 2020; Awong-Taylor et al., 2016, 2018). All of these may contribute to community building and STEM identity development, but additional components of the program would be needed to fulfill all four of the areas identified by Lane (2016). Together, these studies all indicate that a program such as PSI can be made even more powerful when combined with other impactful practices such as summer bridge programs, targeted mentoring/advising, and additional career and research seminars for at-risk students.

### Limitations of study

This study used HS GPA as an indicator of college preparedness in order to draw conclusions regarding the impact of PSI on different student populations. Using HS GPA, college entry exam scores, or some other academic measure to verify the effect of SI on final course grades is a common practice (Dawson et al., 2014). However, as some studies have noted, measures of previous academic achievement do not necessarily account for current motivation levels of students, and higher motivation levels can be expected to result in higher final course grades (Dawson et al., 2014; McCarthy et al, 1997). We do acknowledge that student motivation may be an additional contributing factor to improved course outcomes for PSI attendees and plan to investigate this effect in the future. Current PSI participants are surveyed regarding academic mindset, which includes questions related to motivation. However, this survey was added in Spring 2020, when all PSI sessions were converted to a virtual format due to the COVID-19 pandemic, and therefore the data are not directly comparable to that presented here. McCarthy et al. (1997) additionally argue that college and high school learning environments are not identical, and success in the former does not necessarily guarantee success in the latter. They suggest using performance in common non-SI college courses as a second controlling variable. The range of courses investigated in this study, as well as the varying English and mathematics courses that serve as pre-requisites, unfortunately made that impractical for this analysis.

A further limitation regarding the use of HS GPA as a measure of college preparedness is based on the fact that HS GPA may vary considerably depending on both the academic rigor of the high school and the specific classes that a student took. Allensworth and Clark (2020) recently investigated this effect by examining how well both HS GPA and ACT scores predicted 6-year college graduation rates for students who had graduated from Chicago Public Schools over a 4-year period. They found that HS GPA was overall a strong and consistent predictor of college readiness, and performed much better than ACT scores. However, they also saw that students with the same HS GPA from different high schools could have fairly different college graduation rates. This agrees with a 2010 study by Fletcher and Tienda, which examined GPA and 4-year graduation data from four Texas public universities. They found that students identifying as Black/African American and White, and students identifying as Hispanic/Latino and White, had significantly different GPAs and graduation rates even when controlling for HS GPA. However, these gaps largely disappeared or were even reversed when comparing students from the same high school (Fletcher & Tienda, 2010). Together these reports indicate that while HS GPA is a good general predictor of college success, it may be that in this study, students within each HS GPA group shown in Figs. 6 and 7 are not exactly matched according to previous academic experiences and achievement, if they attended different high schools. Future studies could examine the data for students from the same high school to see if different course outcomes for students identifying with different racial/ethnic groups persist, and how PSI affects students from different high schools.

Despite these limitations, the strong evidence that HS GPA is a good predictor of college readiness, combined with our own data showing the correlation between HS GPA and final course GPA, lends confidence to our assertion that PSI attendees had similar levels of academic preparedness compared to all students enrolled in PSI-supported courses. While PSI participation may not be the sole reason for the higher final course grades of PSI attendees, the evidence here strongly suggests that students directly benefitted from attending PSI sessions, and less prepared students saw a greater benefit than well prepared students.

## Conclusion

We previously showed that the PSI program at GGC is effective at improving course completion rates, despite the modifications necessary to employ this program at a school with multiple sections of small class sizes. Here, the previous work was extended to examine the effects of the PSI program on students identifying with HU groups, who make up over 50% of the GGC student body. Final course grade data were aggregated over five semesters (Spring 2017–Spring 2019) and examined as a function of PSI attendance and students’ self-identified racial/ethnic demographics. Students identifying with HU groups were slightly more likely to attend this direct academic support service compared to students identifying with non-HU groups. While self-selection bias may have been a factor for students identifying as Hispanic/Latino, it did not appear to affect PSI attendance for students identifying with any other racial/ethnic group. Students identifying with both HU and non-HU groups showed a significant effect of PSI attendance on final course GPA. The overall effect of PSI attendance on final course GPA was independent of students’ self-identified race/ethnicity.

The effect of PSI attendance on final course GPA was also examined as a function of both students’ self-identified race/ethnicity and college preparedness, as determined by HS GPA. This analysis showed that PSI participation was most beneficial for the least prepared students, consistent with our previous work. Within each HS GPA group, there were no differences in the overall effect of PSI on final course GPA for students identifying with different racial/ethnic groups. Despite this, we conclude that PSI was in fact disproportionately beneficial for students identifying as Black/African American at GGC. These students benefitted from attending as few as 3–5 PSI sessions, and experienced the largest increase in AB rates and largest decrease in DFW rates with frequent PSI attendance. Students identifying as Black/African American likely experienced the greatest benefit from the program because these students on average entered college less prepared yet were more likely to attend PSI sessions, and were thus more likely to experience the maximum benefit from participation in PSI. This result is particularly encouraging since students identifying as Black/African American make up approximately 33% of STEM majors at GGC.

This work indicates that for institutions that have a significant population of academically underprepared students, PSI is a promising method to assist these students in their introductory STEM courses and hopefully improve their overall retention and progression rates. Since PSI is a modified version of the traditional SI program, this also suggests that other institutions may see similar success even if modifications are made in order to adapt SI to their unique structure and student demographics.

Although this work is encouraging in that it shows a method of helping academically underprepared students, many of whom identify with HU groups, it also reveals that there are still systemic differences in K–12 preparation that lead to inequities in college performance between students identifying with HU vs. non-HU groups. Eliminating these educational disparities at the K–12 level thus appears to be the most worthwhile strategy for ensuring equal opportunities for all students in college courses. While these inequities exist, PSI is a proven method for improving student success rates for underprepared students. However, more comprehensive approaches are needed to equalize educational outcomes for students from all backgrounds.

## Data Availability

The datasets generated and/or analyzed during the current study are not publicly available due to the identifiable nature of the data.

## Abbreviations

GGC: Georgia Gwinnett College
HU: Historically underserved
PSI: Peer Supplemental Instruction
SI: Supplemental instruction
STEM: Science, Technology, Engineering, and Math

## Footnotes

1 Demographic data obtained by GGC did not differentiate between the various sub-categories that may correspond to individuals of Asian descent. We recognize that this is a broad label and may in fact include some populations that are considered HU.

2 A grade of W was given a weight of 0, as a withdrawal means that the student did not successfully complete the course. Therefore, this is not a “true” course GPA since withdrawals are typically not factored into course GPA. However, this more accurately reflects the correlation between PSI participation and successful completion of the course.

3 As shown in Table 2, 10.0% of students attended PSI at all. Due to the large sample size (*N* = 18,590) in Fig. 3, we chose to compare the grades of PSI attendees to those of all enrolled students rather than to those of non-attendees, as isolating the data of non-attendees would have required obtaining sensitive identifying information for such a large number of students. Since the “overall” group is dominated (90.0%) by non-attendees, we believe this comparison still illustrates the effect of attending PSI.
